# Exercício Físico Melhora as Funções das Células Progenitoras Endoteliais em Pacientes com Síndrome Metabólica

**DOI:** 10.36660/abc.20200028

**Published:** 2021-07-15

**Authors:** Qiang Tan, Yang Li, Yao Guo

**Affiliations:** 1 Qinhuangdao First HospitalQinhuangdaoChinaQinhuangdao First Hospital,Qinhuangdao – China

**Keywords:** Células Progenitoras Endoteliais/citologia, Síndrome Metabólica, Exercício, Obesidade, Atividade Física, Inflamação, Óxido Nítrico, Resistência à Insulina, fatores de risco, Doenças Cardiovasculares

## Abstract

**Fundamento:**

As células progenitoras endoteliais (CPEs) desempenham um papel importante na manutenção da função endotelial. A síndrome metabólica (SM) está associada à disfunção das CPEs. Embora o exercício físico tenha um impacto benéfico na atividade das CPEs, seu mecanismo ainda não está completamente esclarecido.

**Objetivo:**

O objetivo deste estudo é investigar os efeitos do exercício físico nas funções das CPEs e os mecanismos subjacentes em pacientes com SM.

**Métodos:**

Os voluntários com SM foram divididos em grupo exercício (n=15) e grupo controle (n=15). Antes e após 8 semanas de treinamento físico, as CPEs foram isoladas do sangue periférico. Foram feitos o ensaio de unidades formadoras de colônias (UFC), o ensaio de formação de tubos, a expressão proteica do óxido nítrico sintase endotelial (eNOS), da fosfatidilinositol-3-quinase (PI3-K) e da proteína quinase B (AKT). Considerou-se um valor de probabilidade <0,05 para indicar significância estatística.

**Resultados:**

Após 8 semanas, o número de UFCs aumentou significativamente no grupo exercício em comparação com o grupo controle (p<0,05). Além disso, observamos uma diminuição significativa do modelo de avaliação da homeostase da resistência à insulina (HOMA-IR), endotelina-1, proteína C reativa de alta sensibilidade e dos níveis de homocisteína no grupo exercício. A intervenção com exercícios também pode aumentar a capacidade de formação de tubos de CPEs e aumentar o nível de fosforilação de eNOS, PI3-K e AKT.

**Conclusão:**

O exercício físico aprimorou as funções das CPEs. O mecanismo pode estar relacionado ao exercício, ativando a via PI3-K/AKT/eNOS.

## Introdução

A síndrome metabólica (SM) compreende um agrupamento de anormalidades como obesidade central, resistência à insulina, dislipidemia e hipertensão.^1^ A SM tem prevalência no mundo. A Federação Internacional de Diabetes (FID) estima que um quarto da população adulta mundial tem SM.^2^ Pacientes com SM têm demonstrado risco aumentado de doença cardiovascular.^3^ Embora a etiologia das complicações vasculares relacionadas à SM não seja totalmente compreendida, a disfunção endotelial pode ser um dos mecanismos possíveis.^4^

As células progenitoras endoteliais (CPEs), originadas da medula óssea, têm a capacidade de circular, se proliferar e se diferenciar em células endoteliais maduras. As CPEs contribuem tanto para a reendotelização quanto para a neoangiogênese, desempenhando um papel vital na manutenção da função endotelial.^5^ Estudos anteriores demonstraram que a síndrome metabólica não apenas diminuiu o nível de CPEs circulantes, mas também prejudicou as funções das CPEs.^6,7^ Alguns estudos^8,9^ relataram que o treinamento físico aeróbio pode melhorar o número de CPEs circulantes em repouso em pessoas saudáveis ou adultos obesos, e as reduções na atividade física reduzem o número de CPEs circulantes.^10^ Embora esses achados sugiram que o exercício aeróbio pode modular as funções das CPEs, o mecanismo é pouco compreendido.

O óxido nítrico (NO) é um importante fator de relaxamento dependente do endotélio.^11^ Sua produção está comumente associada à expressão e atividade da óxido nítrico sintase endotelial (eNOS). Relatou-se que a SM diminuiu a expressão de eNOS e a produção de NO.^12^ O aspecto principal da SM é a resistência à insulina e hiperinsulinemia. Nosso estudo anterior demonstrou que a hiperinsulinemia prejudicou a capacidade de formação de tubos de CPEs por deprimir a fosforilação da eNOS.^13^ Nossa hipótese é que o exercício físico pode ativar a via da eNOS das CPEs e restaurar a função comprometida das CPEs.

O objetivo deste estudo é investigar os efeitos do exercício físico nas funções das CPEs e os mecanismos subjacentes em pacientes com SM.

## Materiais e Métodos

### População do estudo

Os voluntários foram recrutados no centro de exames médicos, o primeiro hospital de Qinhuangdao. Critérios de inclusão: 1. Idade 30–65 anos. 2. Indivíduos com SM. A SM foi definida usando os critérios^14^ do Programa Nacional de Informações sobre o Colesterol — Painel de Tratamento de Adultos III (no mínimo, três critérios baseados em cinco componentes: circunferência abdominal, pressão arterial, glicose sanguínea, triglicerídeos (TG) e colesterol lipoproteína de alta densidade (colesterol HDL).^3^ Os participantes não fizeram exercícios regulares por seis meses. 4. Os participantes não consumiram álcool por dois meses antes deste estudo. Critérios de exclusão: 1. Indivíduos com doença cardiovascular ou doença cerebrovascular. 2. Tabagista. 3. Pacientes com câncer. 4. Usando medicamentos que afetam a função das CPEs, como estatinas, probucol, metformina, bloqueador do receptor de angiotensina. Os voluntários recrutados (n=30) foram divididos aleatoriamente em grupo exercício (n=15) ou grupo controle (n=15). A randomização foi feita com o uso de envelopes lacrados contendo uma sequência de randomização gerada por computador. Baseamos o tamanho da amostra que usamos neste estudo em dados preliminares. Nosso cálculo de potência considerou um aumento de no mínimo 70% nas UFCs de CPEs com um desvio padrão de 40%. Para uma potência de 0,9 (90%), usando um teste t de duas amostras para comparações, foi necessário um tamanho de amostra de pelo menos 8 em cada grupo. O presente estudo foi aprovado pelo Comitê de Ética do Qinhuangdao First Hospital.

### Programa de exercícios

Os voluntários do grupo exercício realizaram um programa de treinos seis dias por semana durante 8 semanas. Fizeram 30 minutos de corrida em esteira mantendo 60% da frequência cardíaca máxima como exercício aeróbio. Também foram submetidos a 30 minutos de exercícios com transpiração, incluindo agachamento, levantamento terra e supino como exercício anaeróbico.^8^ Eles podiam começar a se exercitar a qualquer hora do dia.

### Exames laboratoriais

Variáveis físicas e antropométricas foram medidas no início e após 8 semanas em ambos os grupos. O índice de massa corporal (IMC) foi calculado dividindo-se o peso em quilogramas pela altura ao quadrado em metros. A circunferência abdominal foi medida na altura do umbigo por meio de fita plástica flexível, com os participantes em pé. A pressão arterial foi medida após os participantes terem descansado por 10 minutos (método da ausculta). Foram coletadas amostras de sangue venoso de todos os participantes no início e após 8 semanas (ao término do programa de exercícios, as amostras de sangue foram coletadas 48 horas depois). Mediu-se colesterol total (TC), TG, colesterol HDL, colesterol lipoproteína de baixa densidade (LDL), homocisteína (Hci), glicose e insulina. A RI foi avaliada usando o Modelo de Avaliação da Homeostase da Resistência à Insulina (HOMA-IR) e foi calculada como HOMA-IR (mmol/L×μU/mL) = glicose em jejum (mmol/L) × insulina em jejum (μU/mL)/22,5. As concentrações séricas de NO, ET-1, proteína C reativa de alta sensibilidade (PCR) foram analisadas usando kits ELISA (Zhuocai Biotech, Xangai, China), seguindo as instruções do fabricante para cada kit.

### Cultura de células progenitoras endoteliais

As CPEs foram isoladas e cultivadas, seguindo protocolos previamente descritos.^13^ Resumidamente, coletou-se sangue periférico (15 mL) no início e após o programa de treinamento físico. Células mononucleares (CMNs) foram isoladas e cultivadas em placa de seis poços revestida com fibronectina em meio MCBD/F12 com suplementos (10% FBS, VEGF10 ng/ml, bFGF 10 ng/ml, IGF 10 ng/ml, EGF 10 ng/ml, heparina 10 U/ml e antibióticos) (Gibco) a 37 °C em uma incubadora com 5% de CO_2_. Após mudança de meio no dia 2, o meio foi substituído a cada 3 dias.

### Ensaio de unidade formadora de colônias

Células em forma de paralelepípedo surgiram 5 a 7 dias após o início da cultura de CMN.

Após 12 dias de cultivo, a unidade formadora de colônias (UFC) foi identificada por inspeção visual com microscópio invertido (Leica). Um cluster central rodeado por células emergentes foi reconhecido como uma UFC. Realizou-se ensaio UFC em todos os participantes (grupo exercício, n=15 e grupo controle, n=15).

### Ensaio de formação de tubos

Realizou-se ensaio de formação de tubos para avaliar o potencial angiogênico das CPEs in vitro.^15^ As CPEs foram coletadas e ressuspensas em meio MCBD/DF12 com 2% de soro fetal bovino (FBS). Essas células foram semeadas (50.000 células/poço) em uma placa de cultura de tecidos de 24 poços que havia sido uniformemente revestida com matrigel (BD Labware). As placas semeadas foram incubadas em uma incubadora com 5% de CO2 a 37 ºC durante 4 dias. Os géis foram examinados usando microscopia de contraste de fases (Leica) e utilizou-se o plugin Angiogenesis Analyzer for ImageJ para determinar o comprimento total do segmento tubular, a área total da estrutura tubular e o número de junções de rede em 5 campos selecionados aleatoriamente.

### Análise de western blots

Extraiu-se a proteína total das CPEs com tampão de lise para ensaio de radioimunoprecipitação (Beyotime Biotech, Xangai, China). As concentrações de proteína foram determinadas pelo kit de ensaio BCA (Beyotime Biotech). Amostras iguais de proteínas (40 µg) foram carregadas em cada poço de gel proteico Pierce Precise Protein (Thermo-Fisher, Waltham, MA), tendo sido corridas em tampão de corrida 1 × Tris/HEPES/SDS a 100 V por 1 h. Em seguida, as proteínas foram transferidas para membranas de difluoreto de polivinilideno e bloqueadas com BSA a 5% durante 2 h a 25 °C. As membranas foram então incubadas com anticorpos primários a 4 °C durante a noite (1:1000 em BSA/TBS-T a 1%) e com anticorpos secundários (1:2000 em BSA/TBS-T a 1%) em temperatura ambiente por 2 h. As membranas foram lavadas duas vezes com TBS-T por 10 min antes das incubações e uma vez após as incubações. O complexo ligado foi detectado pelo sistema Odyssey Infrared Imaging System (Li-Cor; Lincoln, NE). As imagens foram analisadas no Image Studio Lite versão 5.2 (LI-COR), para obtenção das intensidades integradas. Os anticorpos primários anti-phospho-Akt-Ser^473^ (1:1.000), anti-Akt (1:1.000), anti-phospho-eNOS-Ser^1177^ (1:1.000), anti-eNOS (1:1.000), anti-β-actin (1:5.000), anti-PI3-K (1:1.000), anti-phospho-PI3-K (1:1.000) e anticorpo anticoelho produzido em cabras conjugado com peroxidase *horseradish* (1:5.000) foram adquiridos da Cell Signaling Technology (Beverly, MA, EUA).

### Análise Estatística

Todas as análises estatísticas foram realizadas com o software SPSS 17 (SPSS Inc., Chicago, IL, EUA). As variáveis contínuas foram expressas como média±desvio padrão. As variáveis categóricas foram expressas em números. As variáveis categóricas foram comparadas pelo teste exato de Fisher. O teste de Shapiro-Wilk foi usado para testar a normalidade da distribuição. As comparações entre variáveis contínuas foram feitas pelo teste t não pareado entre grupos diferentes. E o teste t pareado foi usado para analisar a significância das comparações intergrupo. Considerou-se um valor de probabilidade <0,05 para indicar significância estatística.

## Resultados

### Características físicas dos grupos

As características físicas basais e de 8 semanas são apresentadas na Tabela 1. No início do estudo, as características físicas não eram significativamente diferentes entre os dois grupos. Após 8 semanas de exercício físico, a circunferência abdominal e o IMC diminuíram no grupo exercício, embora não apresentassem diferença estatística em relação ao grupo controle.

**Tabela 1:** – Características dos participantes

	Grupo exercício (n=15)	Grupo controle (n=15)	Valor de p
Idade (anos)	50,71±9,68	50,28±11,34	0,819
Sexo (feminino/masculino)	12/3	11/4	0,664
Altura (cm)	170,91±7,18	170,66±7,46	0,918
Peso (Kg) basal	87,42±11,56	88,22±12,91	0,849
8 semanas	85.41± 10,97	87,51±11,18	0,592
IMC (kg/m^2^) basal	29,86±2,97	30,04±1,99	0,837
8 semanas	29,32±2,59	29.97±1,91	0,415
Circunferência abdominal (cm) basal	96,13±9,72	97,27±10,24	0,746
8 semanas	94,46±9,06	96,94±9,69	0,456
PAS (mmHg) basal	140,53±5,66	135,66±6,36	0,380
8 semanas	138,56±5,91	134,72±5,54	0,111
PAD (mmHg) basal	81,73±8,95	79,22±9,38	0,441
8 semanas	84,86±6,17	82.11± 7,58	0,614

*IMC: índice de massa corporal; PAS: pressão arterial sistólica; PAD: pressão arterial diastólica; PCR: proteína C reativa. O valor de p se refere à comparação entre o grupo exercício e o grupo controle (utilizou-se o teste t pareado ou o teste exato de Fisher).*

### O exercício físico diminuiu a resistência à insulina e o marcador de inflamação

Após 8 semanas, os pacientes do grupo exercício apresentaram menor nível de insulina e HOMA-IR do que os do grupo controle. Esse resultado indicou que o exercício físico pode diminuir a resistência à insulina em pacientes com SM. Os resultados também mostram que marcadores de inflamação como PCR e ET-1 apresentavam-se menores no grupo exercício. O nível de Hci mostrou-se reduzido no grupo exercício. No entanto, não houve diferença significativa de glicose, NO, TG, TC, colesterol LDL e colesterol HDL entre os dois grupos. Para conferir mais detalhes, consulte a Tabela 2.

**Tabela 2:** – Comparação dos parâmetros laboratoriais entre os grupos

	Grupo exercício (n=15)	Grupo controle (n=15)	Valor de p
CT (mmol/L) basal	4.61±1,45	4,39±1,23	0,618
8 semanas	4,24±1,24	4,25±1,34	0,980
TG (mmol/L) basal	2,19±0,71	1,92±0,89	0,352
8 semanas	2,24±0,83	2,05±0,87	0,515
Colesterol LDL (mmol/L) basal	2,58±1,02	2,51±0,89	0,623
8 semanas	2,47±0,91	2,61±0,95	0,753
Colesterol HDL (mmol/L) basal	1,03±0,21	1,06±0,18	0,623
8 semanas	1,06±0,12	1,05±1,05	0,763
Hci (μmol/L) basal	14,84±6,99	15,13±4,68	0,774
8 semanas	11,31±3.07^**#**^	14,91±2,96	0,020
PCR-us (mg/L) basal	3,44 ±2,72	4.98± 3,22	0,338
8 semanas	1,75±0.94^**#**^	3,88±2,13	0,047
Glicose (mmol/L) basal	7,05±1,39	7,14±2,95	0,536
8 semanas	6,51±3,95	6,59±2,02	0,374
Insulina (UI/mL) basal	7,49±4,45	6,67±3,12	0,746
8 semanas	5,48±2.96^**#**^	7,59±3,89	0,039
HOMA-IR basal	2.91± 1,91	2,76±0,61	0,645
8 semanas	2,08±1.25^**#**^	2,81±0,76	0,037
ON (μmol/L) basal	137.41± 94,17	154,82±87,12	0,585
8 semanas	141,92±40,62	167,15±119,89	0,139
ET-1 (μmol/L) basal	2,71±1,18	2,61 ±1,28	0,998
8 semanas	1,62±0.66^**#**^	2,51±1,17	0,041

*HOMA-IR: modelo de avaliação da homeostase da resistência à insulina; CT: colesterol total; TG: triglicerídeos; LDL: lipoproteína de baixa densidade; HDL: lipoproteína de alta densidade; PCR-us: proteína C reativa ultrassensível. Hci: homocisteína; ON: óxido nítrico; ET-1: endotelina-1. O valor de p se refere à comparação entre grupos diferentes (teste t pareado). #p<0,05 em comparação com os valores basais (teste t pareado).*

### O exercício físico aumentou a formação de colônias de CPEs

**Figura 1:**
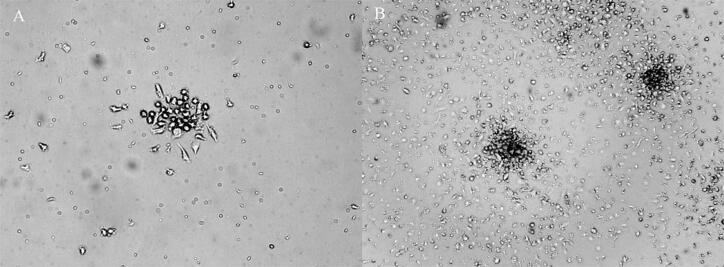
– Colônia de CPEs cultivadas. A) Seis dias após a cultura, apareceram CPEs em formato de paralelepípedo (aumento de 100 ×). B) Doze dias após a cultura, colônia de CPEs (aumento de 50 ×).

Surgiram colônias de CPEs 5 a 7 dias após o início da cultura de CMN. A colônia exibiu morfologia de “paralelepípedo” e padrão de crescimento em monocamada (Figura 1).

**Figura 2:**
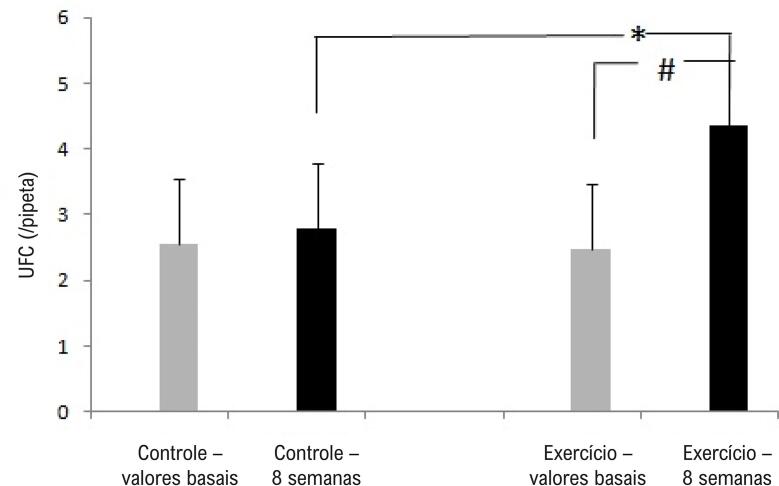
– O exercício físico aumentou as UFCs de CPEs. Aumento de UFCs de CPEs no grupo exercício após 8 semanas. *p<0,05 comparação entre o grupo exercício e o grupo controle; #p<0,05 comparação com os valores basais.

Conforme mostrado na Figura 2, após 8 semanas de exercício físico, o número de UFCs de CPEs apresentou-se maior no grupo exercício do que no grupo controle (p<0,05).

### O exercício físico melhorou a capacidade de formação de tubos de CPEs

**Figura 3:**
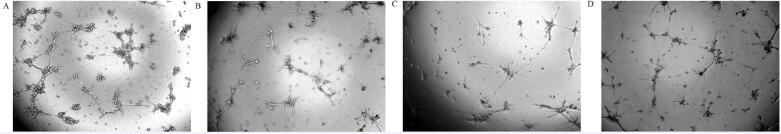
– O exercício físico melhorou a capacidade de formação de tubos das CPEs. A: grupo controle no início do estudo; B: grupo controle após 8 semanas; C: grupo exercício no início do estudo; D: grupo exercício após 8 semanas. A figura mostra redes tubulares formadas por CPEs em matrigel. Após 8 semanas, as CPEs do grupo exercício tinham redes tubulares mais longas e melhores.

Como mostra a Figura 3, as CPEs formaram redes tubulares no matrigel. O comprimento total da rede tubular, a área total da rede tubular e o número de junções foram medidos pelo sistema de análise de imagem. Após 8 semanas de exercício físico, as CPEs do grupo exercício demonstraram formação de rede aumentada, em comparação com o grupo controle (p<0,05) (ver tabela 3).

**Tabela 3:** – Comparação da capacidade de formação de tubos entre o grupo exercício e o grupo controle

	Grupo exercício (n=15)	Grupo controle (n=15)	Valor de p
Comprimento (μm/campo) basal	2913.20± 662,05	2512,01±829,46	0,154
8 semanas	3982,67 ±832.94^#^	2713,33±705,57	0,000
Área (μm^2^/campo) basal	278,60±93,34	274,86±95,57	0,915
8 semanas	440,66±100,74^#^	276.01± 72,88	0,000
Junção (/campo) basal	8,93±3,59	9,06±2,84	0,911
8 semanas	12,60±2.74^#^	8,93±2,08	0,001

*O valor de p se refere à comparação entre o grupo exercício e o grupo controle (utilizou-se o teste t pareado). #p<0,05 em comparação com os valores basais (utilizou-se teste t pareado).*

### O exercício físico aumentou a fosforilação de PI3-K/Akt/eNOS

**Figura 4:**
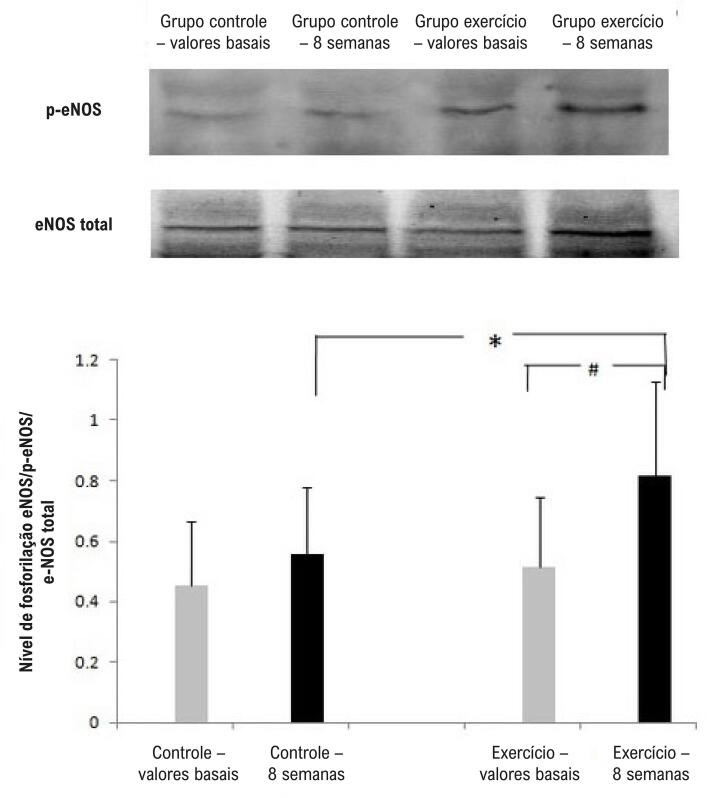
– Western blot da eNOS. O exercício físico pode aumentar o nível de fosforilação da eNOS de CPEs. N=4, *p<0,05 comparação entre o grupo exercício e o grupo de controle; #p<0,05 comparação com os valores basais.

**Figura 5:**
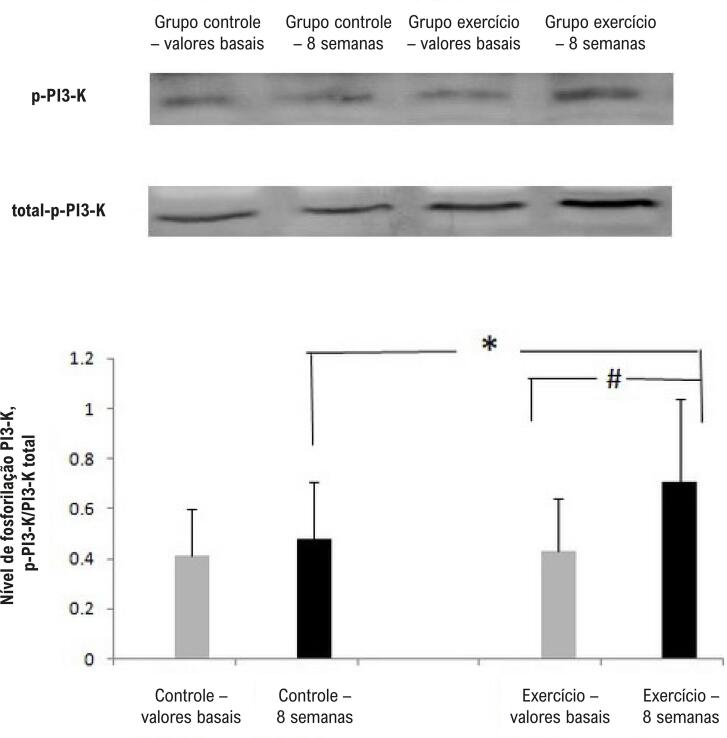
– Western blot da PI3-K. Os níveis de fosforilação de PI3-K aumentaram no grupo exercício após 8 semanas. N=4, *p<0,05 comparação entre o grupo exercício e o grupo de controle; #p<0,05 comparação com os valores basais.

**Figura 6:**
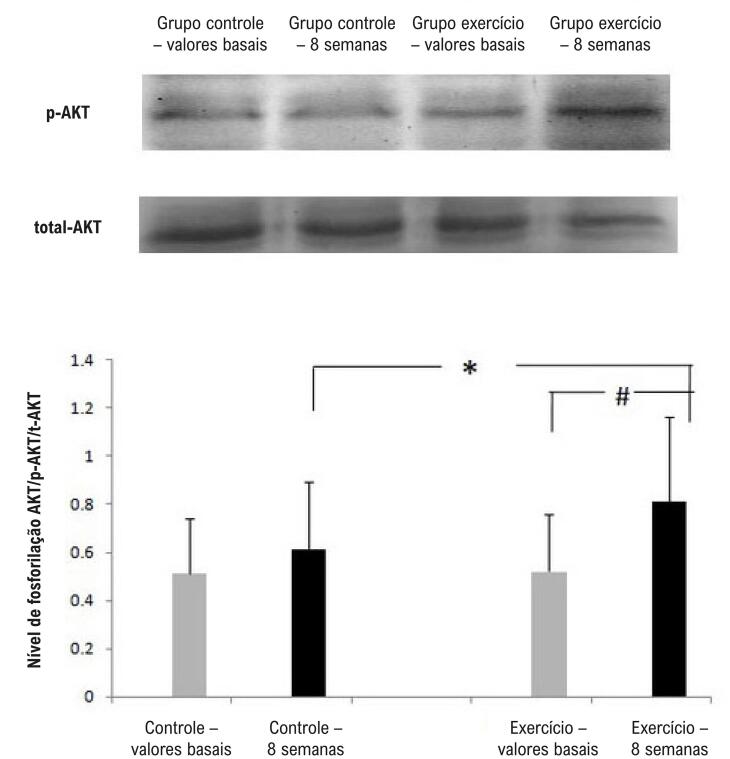
– Western blot da PI3-K. Os níveis de fosforilação da AKT aumentaram no grupo exercício após 8 semanas. N=4, *p<0,05 comparação entre o grupo exercício e o grupo de controle; #p<0,05 comparação com os valores basais.

Não houve diferença na expressão da proteína fosforilada de PI3-K, AKT e eNOS no início do estudo. Após o programa de 8 semanas, conforme mostram as figuras 4–6, o exercício físico aumentou o nível de fosforilação de PI3-K, AKT e eNOS em CPEs em comparação com o grupo controle (p<0,05).

## Discussão

O presente estudo demonstrou que oito semanas de exercício físico podem melhorar as funções das CPEs em pacientes com SM. O mecanismo pode estar relacionado ao exercício de ativação da via PI3-K/AKT/eNOS.

As CPEs estão envolvidas na neovasculogênese e na manutenção da integridade vascular. Um estado alterado de CPEs circulantes representa um marcador de disfunção endotelial.^3^ De fato, alguns estudos indicaram que o número de CPEs circulantes é um indicador independente da saúde cardiovascular.^16,17^ Nosso estudo anterior demonstrou que os pacientes com SM tiveram um nível significativamente reduzido de CPEs circulantes em comparação com os do grupo controle saudáveis.^6^ O estudo de Jialal et al. mostra que CPEs de indivíduos com SM apresentam capacidade clonogênica significativamente comprometida, unidades formadoras de colônias reduzidas e capacidade comprometida de incorporação em estruturas tubulares.^7^ O exercício físico é um importante estímulo fisiológico para mobilizar as CPEs em indivíduos saudáveis.^18^ Como sabemos, as CPEs são um grupo heterogêneo de células.^19^ Existem dois tipos de CPEs circulantes: as CPEs *early* e as células endoteliais maduras (OEC, do inglês *outgrowth endothelial cell*). As CPEs *early* são células fusiformes e não têm capacidade de formar colônias. Os OECs têm potencial de formação de colônias e aparência de paralelepípedos. Os OECs têm maior capacidade aderente e de formação tubular. São mais importantes na angiogênese do que as CPEs *early.*^8^ No presente estudo, observamos colônias de células em forma de paralelepípedo, mas não de células fusiformes. Nossos resultados indicaram que o exercício físico aumentou a UFC de CPEs em pacientes com SM.

Os resultados deste estudo indicaram que o exercício físico melhorou a capacidade de formação de tubos das CPEs. Esses achados estão de acordo com estudos anteriores. Silva et al. apontaram que o exercício físico pode preservar a função endotelial em camundongos obesos.^11^ Choi et al.^8^ demonstraram que o exercício regular aumentou a UFC de CPEs em indivíduos saudáveis.^8^ O estudo de Landers-Ramos et al. mostrou que 10 dias de exercício físico aeróbio foram suficientes para aumentar o número de células CD34^+^/KDR^+^ e KDR^+^ e melhorar a dilatação fluxo-mediada em idosos previamente sedentários.^20^ Embora esses estudos tenham demonstrado os benefícios do exercício físico, seu mecanismo ainda não está elucidado. O presente estudo mostra que o exercício físico elevou a fosforilação da eNOS das CPEs. Como sabemos, a biodisponibilidade de óxido nítrico é um importante regulador da reatividade vascular e da função endotelial. Além de promover o vasorrelaxamento, regula a angiogênese em resposta à isquemia tecidual.^12^ A diabetes pode prejudicar a função das CPEs modificando os mecanismos relacionados ao óxido nítrico. Chen et al. apontaram que a fosforilação da eNOS e a produção de NO em meio de cultura de CPEs se reduziu quando as células foram incubadas em 25 mmol/L de glicose em comparação com 5 mmol/L de glicose.^21^ A via PI3-K/Akt/eNOS é uma via clássica para promover a produção de NO e desempenha um papel vital na regulação da angiogênese de CPEs. Nosso estudo anterior constatou que a hiperinsulinemia deprimiu a fosforilação da eNOS por meio da depressão da via PI3-K/Akt, associada ao comprometimento da capacidade de formação de tubos das CPEs.^13^ O presente estudo indicou que o exercício físico pode ativar a via PI3-K/Akt/eNOS em pacientes com SM. Como resultado, o exercício físico restaurou o comprometimento da capacidade de formação de tubos de CPEs em pacientes com SM.

A função endotelial depende do delicado equilíbrio entre vasodilatadores e vasoconstritores.^22^ Como um forte vasoconstritor, a ET-1 elevada desempenha um papel fundamental no desenvolvimento da disfunção endotelial. Nossos resultados demonstraram que o exercício reduziu as concentrações circulantes de ET-1 na SM. Esse achado está de acordo com o estudo de Dow et al.^23^ A redução da ET-1 pode ser um mecanismo importante subjacente à melhora induzida pelo exercício na vasodilatação dependente do endotélio.

## Limitações

Em primeiro lugar, nós não exploramos a secreção parácrina de CPEs. Embora tenhamos detectado citocinas como NO, PCR, Hci e ET-1 em circulação, não detectamos citocinas em meio de cultura de CPEs. Em segundo lugar, não medimos a dilatação fluxo-mediada (FMD) em pacientes com SM. A FMD é um tipo de método ubíquo para avaliar a função endotelial. Mas não medimos a FMD porque a FMD não tem sensibilidade suficiente na SM. Em terceiro lugar, a síndrome metabólica pode induzir apoptose de CPEs. Porém, neste estudo, não detectamos apoptose de CPEs. Em quarto lugar, os mecanismos relacionados ao exercício físico são muito complexos. O exercício pode influenciar a inflamação e o estresse oxidativo. Os resultados deste estudo mostram que o exercício físico diminuiu o nível circulante de PCR e Hci, indicando que o exercício físico pode diminuir a inflamação e o estresse oxidativo em pacientes com SM. Mas ainda não sabíamos a correlação entre inflamação e disfunção de CPEs.

## Conclusões

Em conclusão, este estudo demonstrou que oito semanas de exercício físico melhoraram as funções das CPEs em pacientes com SM. O mecanismo pode estar relacionado ao exercício de ativação da via PI3-K/AKT/eNOS. Este estudo também revelou que o exercício deprimiu a inflamação e o estresse oxidativo em pacientes com SM. Mas não sabíamos a correlação de inflamação e disfunção de CPEs.
