# Supplementary material for: Exercício Físico Melhora as Funções das Células Progenitoras Endoteliais em Pacientes com Síndrome Metabólica
Source: Arq Bras Cardiol. 2021 Jul 15;117(1):108–17. [Article in Portuguese] doi: 10.36660/abc.20200028 (PMC8294748; doi:10.36660/abc.20200028)
Supplement: Translation

# Translation: Exercise Training Improves Functions of Endothelial Progenitor Cells in Patients with Metabolic Syndrome

**Authors:** Qiang Tan, Yang Li, Yao Guo

**Affiliations:** 1 ChinaQinhuangdao First Hospital, Qinhuangdao – China

**Keywords:** Endothelial Progenitor Cells/citology, Metabolic Syndrome, Exercise, Obesity, Physical Activity, Inflammation, Nitric Oxide, Insulin Resistance, Risk Factors, cardiovascular Diseases

## Abstract

**Background:**

Endothelial progenitor cells (EPCs) play an important role in maintaining endothelial function. Metabolic syndrome (MetS) is associated with EPC dysfunction. Although physical exercise has a beneficial impact on EPC activity, its mechanism is not completely clear yet.

**Objective:**

The purpose of this study is to investigate the effects of physical exercise on the functions of EPCs and the underlying mechanisms in patients with MetS.

**Methods:**

Volunteers with MetS were divided into exercise group (n=15) and control group (n=15). Before and after 8 weeks exercise training, EPCs were isolated from peripheral blood. Colony forming unit (CFU) assay, tube-formation assay, the protein expression of endothelial nitric oxide synthase (eNOS), phosphatidylinositol-3-kinase (PI3-K) and protein kinase B (AKT) were determined. A probability value <0.05 was considered to indicate statistical significance.

**Results:**

After 8 weeks, the number of CFUs was significantly increased in the exercise group compared to the control group (p<0.05). In addition, we observed a significant decrease of homeostasis model assessment for insulin resistance (HOMA-IR), endothelin-1, high-sensitive C-reactive protein, and homocysteine levels in the exercise group. Exercise intervention could also enhance tube-formation capacity of EPCs and increase phosphorylation level of eNOS, PI3-K and AKT.

**Conclusion:**

Physical exercise enhanced the functions of EPCs. The mechanism may be related to exercise, activating the PI3-K/AKT/eNOS pathway.

## Introduction

Metabolic syndrome (MetS) comprises a cluster of abnormalities, including central obesity, insulin resistance, dyslipidemia and hypertension.^1^ MetS is prevalent in the world. The International Diabetes Federation (IDF) estimates that one-quarter of the world’s adult population has MetS.^2^ Patients with MetS have been shown to have an increased risk for cardiovascular disease.^3^ Although the etiology of MetS related to vascular complications is not fully understood, endothelial dysfunction maybe one of the possible mechanisms.^4^

Endothelial progenitor cells (EPCs), originating in the bone marrow, have the capacity to circulate, proliferate, and differentiate into mature endothelial cells. EPCs contribute to both reendothelialization and neoangiogenesis, thereby EPCs play a vital role in maintaining endothelial function.^5^ Previous studies demonstrated that metabolic syndrome did not only decrease the levels of circulating EPCs, but also impaired the functions of EPCs.^6,7^ Some studies^8,9^ reported that aerobic exercise training could improve the number of resting circulating EPCs in healthy people or obese adults, and reductions in physical activity reduce the number of circulating EPCs.^10^ Although these findings suggest that aerobic exercise may modulate EPC functions, the mechanism is poorly understood.

Nitric oxide (NO) is an important endothelium-dependent relaxant factor^11^ Its production is commonly associated with expression and activity of endothelial nitric oxide synthase (eNOS). It was reported that MetS decreased expression of eNOS and production of NO.^12^ The core of MetS is insulin resistance and hyperinsulinemia. Our previous study demonstrated that hyperinsulinemia impaired tube-formation ability of EPCs by depressing eNOS phosphorylation.^13^ We hypothesized that exercise training may activate the eNOS pathway of EPCs and restore impaired function of EPCs.

The purpose of this study is to investigate the effects of physical exercise on the EPC functions and the underlying mechanisms in patients with MetS.

## Materials and Methods

### Study population

Volunteers were recruited from a medical examination center, the first hospital of Qinhuangdao. Inclusion criteria: 1. Aged 30–65. 2. Subjects with MetS. MetS was defined using the criteria^14^ of the National Cholesterol Education Program — Adult Treatment Panel-III (at least three criteria based on five components: waist circumference, blood pressure, blood glucose, triglycerides (TG), and high-density lipoprotein cholesterol (HDL-C).^3^ Subjects had no regular exercise for six months. 4. Subjects had not consumed alcohol for two months prior to this study. Exclusion criteria: 1. Subjects with cardiovascular disease or cerebrovascular disease. 2. Smokers. 3. Patients with cancer. 4. On medications known to affect EPC function, such as statins, probucol, metformin, angiotensin receptor blockers. Recruited volunteers (n=30) were randomly divided into exercise group (n=15) or control group (n=15). Randomization was done with sealed envelopes containing a computer-generated randomization sequence. Sample size was based on *preliminary data*. Our power calculation assumed at least a 70% increase in EPC-CFUs with a standard deviation of 40%. For a required power of 0.9 (90%), using a two-sample t-test for comparisons, a sample size of at least 8 in each group was required. This study was approved by the ethics committee of the Qinhuangdao First Hospital.

### Exercise training program

Volunteers from the exercise group performed a training program six days per week for 8 weeks. As an aerobic exercise, they ran on a treadmill for 30 minutes while maintaining 60% of the maximum heart rate. As an anaerobic exercise, they completed 30-minute training wet with perspiration including, squats, deadlifts and bench press.^8^ They could start exercise at any time of the day.

### Laboratory measurements

Physical and anthropometric variables were measured at baseline and after 8 weeks in both groups. Body mass index (BMI) was calculated as weight in kilograms divided by the square of the height in meters. Waist circumference was measured at the umbilicus level using a flexible plastic tape with the participants in the standing position. Blood pressure was measured after participants had rested for 10 minutes (auscultatory method). Venous blood samples were collected from all participants at baseline and at 8 weeks (when the exercise program finished, blood samples were collected 48 hours later). Total cholesterol (TC), TG, HDL-C, low-density lipoprotein cholesterol (LDL-c), homocysteine (HCY), glucose, and insulin were measured. IR was evaluated using the Homeostasis Model of Assessment of Insulin Resistance (HOMA-IR) and was calculated as HOMA-IR (mmol/L×μU/mL) = glicose em jejum (mmol/L) × insulina em jejum (μU/mL)/22,5. Serum concentrations of NO, ET-1, high-sensitivity C-reactive protein (hsCRP) were analyzed using ELISA kits (Zhuocai Biotech, Shanghai, China), following the manufacturer’s instructions for each kit.

### Endothelial progenitor cells culture

EPCs were isolated and cultured, following previously described protocols.^13^ Peripheral blood (15 mL) was withdrawn at baseline and after the exercise training program. Mononuclear cells (MNCs) were isolated and cultured on a fibronectin-coated six-well chamber in MCBD/F12 medium with supplements (10% FBS, VEGF10 ng/ml, bFGF 10 ng/ml, IGF 10 ng/ml, EGF 10 ng/ml, heparin 10 U/ml, and antibiotics) (Gibco) at 37 °C in a 5% CO_2_ incubator. After changing the medium on day 2, the medium was replaced every 3 days.

### Colony-forming unit assay

Cobblestone-shaped cells emerged 5 to 7 days after start of the MNC culture.

After 12 days of culture, the colony-forming unit (CFU) was identified by visual inspection with an inverted microscope (Leica). A central cluster surrounded by emerging cells was recognized as a CFU. The CFU assay was performed in all subjects (exercise group, n=15 and control group, n=15).

### Tube-formation assay

Tube-formation assay was performed to assess the angiogenic potential of EPCs in vitro.^15^ EPCs were collected and resuspended in MCBD/DF12 medium with 2% FBS. These cells were seeded (50.000 cells/well) in a 24-well tissue culture plate, which had been evenly coated with matrigel (BD Labware). Seeded cells were incubated at 37 °C in a 5% CO_2_ incubator for 4 hours. Gels were examined using phase-contrast microscopy (leica), and the Angiogenesis Analyzer ImageJ plugin was used to determine the total tube-like segment length, the total area of the tubular structure and the number of network junctions in 5 randomly selected fields.

### Western blot analysis

The total protein of EPCs was extracted with radioimmunoprecipitation assay lysis buffer (Beyotime Biotech, Shanghai, China). Protein concentrations were determined by BCA assay kit (Beyotime Biotech). Equal protein samples (40 µg) were loaded into each well of Pierce Precise Protein gel (Thermo-Fisher, Waltham, MA) and were run in 1 × Tris/HEPES/SDS running buffer at 100 V for 1 h. Proteins were then transferred to polyvinylidene difluoride membranes and blocked with 5% BSA for 2 h at 25 °C. Membranes were then incubated with primary antibodies at 4 °C overnight (1:1000 in 1% BSA/TBS-T) and with secondary antibodies (1:2000 in 1% BSA/TBS-T) at room temperature for 2 h. Membranes were washed two times with TBS-T for 10 min before incubations and once after incubations. The bound complex was detected using the Odyssey Infrared Imaging System (Li-Cor; Lincoln, NE). The images were analyzed using Image Studio Lite version 5.2 (LI-COR) to obtain the integrated intensities. Primary antibodies including anti-phospho-Akt-Ser^473^(1:1,000), anti-Akt (1:1,000), anti-phospho-eNOS-Ser^1177^(1:1,000), anti-eNOS (1:1,000), anti-β-actin (1:5,000), anti- PI3-K (1:1,000), anti-phospho-PI3-K (1:1,000) and horseradish peroxidase-conjugated goat anti-rabbit IgG (1:5,000) were purchased from Cell Signaling Technology (Beverly, MA, USA).

### Statistical Analysis

All statistical analyses were performed by SPSS 17 (SPSS, Chicago, IL, USA). Continuous variables were expressed as mean±standard deviation. Categorical variables were expressed as numbers. Categorical variables were compared with the Fisher’s exact test. The Shapiro-Wilk test was used to test for normality of distribution. Comparisons between continuous variables were made by unpaired t test between different groups. And paired t test was used to analyze the significance of within-group comparisons. A probability value <0.05 was considered to indicate statistical significance.

## Results

### Physical characteristics of both groups

Baseline and 8-week physical characteristics are presented in Table 1. At baseline, the physical characteristics were not significantly different between the two groups. After 8 weeks of exercise training, waist circumference and BMI were decreased in the exercise group, although they had no statistical difference compared to the control group.

**Table 1:** – Participant characteristics

	Grupo exercício (n=15)	Grupo controle (n=15)	Valor de p
Age (years)	50.71±9.68	50.28±11.34	0.819
Gender (male/female)	12/3	11/4	0.664
Height (cm)	170.91±7.18	170.66±7.46	0.918
Baseline weight (Kg)	87.42±11.56	88.22±12.91	0.849
8 weeks	85.41± 10.97	87.51±11.18	0.592
Baseline BMI (kg/m^2^)	29.86±2.97	30.04±1.99	0.837
8 weeks	29.32±2.59	29.97±1.91	0.415
Baseline waist circumference (cm)	96.13±9.72	97.27±10.24	0.746
8 weeks	94.46±9.06	96.94±9.69	0.456
Baseline SBP (mmHg)	140.53±5.66	135.66±6.36	0.380
8 weeks	138.56±5.91	134.72±5.54	0.111
Baseline DBP (mmHg)	81.73±8.95	79.22±9.38	0.441
8 weeks	84.86±6.17	82.11± 7.58	0.614

*BMI: body mass index; SBP: systolic blood pressure; DBP: diastolic blood pressure. p value refers to comparison between exercise group and control group (unpaired t test or Fisher’s exact test were used).*

### Exercise training decreased insulin resistance and inflammation marker

After 8 weeks, patients from the exercise group had lower levels of insulin and HOMA-IR than those in the control group. This result indicated that exercise training could decrease insulin resistance in patients with MetS. The results also show that inflammation markers, including CRP and ET-1, were decreased in the exercise group. HCY were decreased in the exercise group. However, there were no significant differences in glucose, NO, TG, TC, LDL-c and HDL-c between the two groups. For more details, see Table 2.

**Table 2:** – Comparison of laboratory parameters between groups

	Exercise group (n=15)	Control group (n=15)	p value
Baseline TC (mmol/L)	4.61±1.45	4.39±1.23	0.618
8 weeks	4.24±1.24	4.25±1.34	0.980
Baseline TG (mmol/L)	2.19±0.71	1.92±0.89	0.352
8 weeks	2.24±0.83	2.05±0.87	0.515
Baseline LDL-C (mmol/L)	2.58±1.02	2.51±0.89	0.623
8 weeks	2.47±0.91	2.61±0.95	0.753
Baseline HDL-C (mmol/L)	1.03±0.21	1.06±0.18	0.623
8 weeks	1.06±0.12	1.05±1.05	0.763
Baseline HCY (μmol/L)	14.84±6.99	15.13±4.68	0.774
8 weeks	11.31±3.07^**#**^	14.91±2.96	0.020
Baseline Hs-CRP (mg/L)	3.44 ±2.72	4.98± 3.22	0.338
8 weeks	1.75±0.94^**#**^	3.88±2.13	0.047
Baseline glucose (mmol/L)	7.05±1.39	7.14±2.95	0.536
8 weeks	6.51±3.95	6.59±2.02	0.374
Baseline insulin (UI/mL)	7.49±4.45	6.67±3.12	0.746
8 weeks	5.48±2.96^**#**^	7.59±3.89	0.039
Baseline HOMA-IR	2.91± 1.91	2.76±0.61	0.645
8 weeks	2.08±1.25^**#**^	2.81±0.76	0.037
Baseline NO (μmol/L)	137.41± 94.17	154.82±87.12	0.585
8 weeks	141.92±40.62	167.15±119.89	0.139
Baseline ET-1 (μmol/L)	2.71±1.18	2.61 ±1.28	0.998
8 weeks	1.62±0.66^**#**^	2.51±1.17	0.041

*HOMA-IR: homeostasis model assessment for insulin resistance; TC: total cholesterol; TG: triglycerides; LDL-C: low-density lipoprotein cholesterol; HDL-C: high-density lipoprotein cholesterol; Hs-CRP: high-sensitive C-reactive protein; HCY: homocysteine; NO: nitric oxide; ET-1: endothelin-1. p value refers to comparison between different groups (unpaired t test). #p<0.05 compared with baseline (paired t test).*

### Exercise training increased EPC colony formation

Colonies of EPC emerged 5 to 7 days after start of MNC culture. The colony exhibited “cobblestone” morphology and monolayer growth pattern (Figure 1).

**Figure 1:** – Figure 1 — Colony of cultured EPCs. A) 6 days after culture, cobblestone-shaped EPCs appeared (100 × magnification). B) 12 days after culture, colony of EPCs (50 × magnification).

As shown in Figure 2, after 8 weeks exercise training, the number of EPC-CFUs was bigger in the exercise group than in the control group (p<0.05).

**Figure 2:** – Exercise training increased EPC-CFU. EPC-CFU was increased in the exercise group after 8 weeks. * p<0.05 comparison between exercise group and control group; #p<0.05 compared with baseline.

## Exercise training improved tube-formation ability of EPCs

As shown in Figure 3, EPCs formed tubular networks on matrigel. Total length of tubular network, total area of tubular network and number of junctions were measured by image analysis system. After 8 weeks exercise training, EPCs in the exercise group demonstrated increased network formation, compared with the control group (p<0.05) (see table 3).

**Figure 3:** – Exercise training improved tube-formation ability of EPCs. A: control group at baseline; B: control group after 8 weeks; C: exercise group at baseline; D: exercise group after 8 weeks. The figure show that EPCs formed tubular networks on matrigel. After 8 weeks, exercise group EPCs had longer and better tubular networks.

**Table 3:** – Comparison of tube-formation ability between the exercise group and control group

	Exercise group (n =15)	Control group (n = 15)	p value
Baseline length (μm/field)	2913.20± 662.05	2512.01±829.46	0.154
8 weeks	3982.67 ±832.94^#^	2713.33±705.57	0.000
Baseline area (μm^2^/field)	278.60±93.34	274.86±95.57	0.915
8 weeks	440.66±100.74^#^	276.01± 72.88	0.000
Baseline junction (/field)	8.93±3.59	9.06±2.84	0.911
8 weeks	12.60±2.74^#^	8.93±2.08	0.001

*p value refers to comparison between exercise group and control group (unpaired t test was used). #p<0.05 compared with baseline (paired t test was used).*

### Exercise training increased PI3-K/Akt /eNOS phosphorylation

There were no differences of phosphorylated protein expression of PI3-K, AKT and eNOS at baseline. After 8 weeks, as shown in figures 4–6, exercise training increased phosphorylation level of PI3-K, AKT and eNOS in EPCs compared with THE control group (p<0.05).

**Figure 4:** – Western blot of eNOS. Exercise training could increase eNOS phosphorylation level of EPCs. N=4, *p<0.05 comparison between exercise group and control group; #p<0.05 compared with baseline.

**Figure 5:** – Western blot da PI3-K. Os níveis de fosforilação de PI3-K aumentaram no grupo exercício após 8 semanas. N=4, *p<0,05 comparação entre o grupo exercício e o grupo de controle; #p<0,05 comparação com os valores basais.

**Figure 6:** – Western blot of PI3-K. AKT phosphorylation levels were increased in the exercise group after 8 weeks. N=4, *p<0.05 comparison between exercise group and control group; #p<0.05 compared with baseline.

## Discussion

The present study demonstrated that eight weeks of exercise training could improve EPC functions in patients with MetS. The mechanism may be related to exercise activating the PI3-K/AKT/eNOS pathway.

EPCs are involved in neovasculogenesis and maintenance of vascular integrity. An altered status of circulating EPCs represents a marker of endothelial dysfunction.^3^ In fact, some studies indicated that the number of circulating EPCs is an independent indicator of overall cardiovascular health.^16,17^ Our previous study demonstrated that patients with MetS had significantly decreased levels of circulating EPCs compared with healthy controls.^6^ A study by Jialal et al. found that EPCs from MetS subjects presented significantly impaired clonogenic capacity, decreased colony-forming units, and impaired capacity to incorporate into tubular structures.^7^ Exercise training is an important physiological stimulus to mobilize EPCs in healthy individuals.^18^ As we know, EPCs are a heterogeneous group of cells.^19^ There are two types of EPCs in circulation: early EPCs and outgrowth endothelial cells (OEC). Early EPCs are spindle-shaped cells and have no colony-forming ability. OECs have colony-forming potential and cobblestone shape. OECs have higher adhesive capacity and tubular forming capacity, and are more important in angiogenesis than early EPCs.^8^ In the current study, we observed a colony of cobblestone-shape cells but not spindle-shape cells. Our findings indicated that exercise training increased CFUs of EPCs in patients with MetS.

The results of this study indicated that exercise training improved the tube-formation ability of EPCs. These findings are consistent with previous studies. Silva et al. reported that exercise training could preserve endothelial function in obese mice.^11^ Choi et al.^8^ demonstrated that regular exercise increased EPC-CFUs in healthy individuals.^8^ A study by Landers-Ramos et al. showed that 10 days of aerobic exercise training was sufficient to increase CD34^+^/KDR^+^ and KDR^+^ cells number and improve flow-mediated dilation in previously sedentary older adults.^20^ Although these studies have demonstrated the benefits of exercise training, its mechanism is not elucidated yet. The current study shows that exercise training elevated phosphorylation of eNOS of EPCs. As we know, nitric oxide bioavailability is an important regulator of vascular reactivity and endothelial function. It does not only promote vasorelaxation but also regulates angiogenesis in response to tissue ischemia.^12^ Diabetes may impair EPC function by modifying nitric oxide-related mechanisms. Chen et al. found that eNOS phosphorylation and NO production in EPC culture media was reduced when cells were incubated in 25 mmol/L glucose compared with 5 mmol/L glucose.^21^ The PI3-K/Akt /eNOS pathway is a classical pathway to promote NO production and plays a vital role in regulating angiogenesis of EPCs. Our previous study testified that hyperinsulinemia depressed eNOS phosphorylation by depressing the PI3-K/Akt pathway, which was associated with the impaired tube-formation ability of EPCs.^13^ The current study indicated that exercise training could activate PI3-K/Akt /eNOS pathway in patients with MetS. As a result, exercise training restored impaired tube-formation ability of EPCs in patients with MetS.

Endothelial function depends on the delicate balance between vasodilators and vasoconstrictors.^22^ As a strong vasoconstrictor, high ET-1 plays a key role in the development of endothelial dysfunction. Our results demonstrated that exercise reduced circulating concentrations of ET-1 in MetS. This finding was consistent with a study by Dow et al.^23^ Reduction of ET-1 may be an important mechanism underlying the exercise-induced improvement in endothelium-dependent vasodilator function.

## Limitations

Firstly, we did not explore paracrine secretion of EPCs. Although we detected cytokines including NO, CRP, HCY and ET-1 in circulation, we did not detect cytokines in the EPC culture medium. Secondly, we did not measure flow-mediated dilation (FMD) in patients with MetS. FMD is a kind of ubiquitous method to evaluate endothelial function. But we did not measure FMD because FMD has not enough sensibility in MetS. Thirdly, metabolic syndrome may induce EPC apoptosis. But in this study, we did not detect apoptosis of EPCs. Fourthly, exercise training-related mechanisms are very complex. Exercise may influence inflammation and oxidative stress. Results of this study show that exercise training decreased circulating levels of CRP and HCY, which indicated that exercise training could depress inflammation and oxidative stress in patients with MetS. But we still did not know the correlation of inflammation and EPC dysfunction.

## Conclusions

In conclusion, this study demonstrated that eight weeks of exercise training improved EPC functions in patients with MetS. The mechanism may be related to exercise activating the PI3-K/AKT/eNOS pathway. This study also revealed that exercise depressed inflammation and oxidative stress in patients with MetS. But we did not know the correlation of inflammation and EPC dysfunction.
